# Effects of Different Proportions of Corn Silage and Ramie Silage on In Vitro Rumen Fermentation Characteristics and Methane Production

**DOI:** 10.3390/ani16081250

**Published:** 2026-04-18

**Authors:** Honghui Qi, Cheng Gao, Zhicai Li, Duanqin Wu

**Affiliations:** 1 Institute of Bast Fiber Crops, Chinese Academy of Agricultural Sciences, Changsha 410205, China; 15714755547@163.com (H.Q.); gaochenger1122@163.com (C.G.); 2 Hunan Institute of Animal Science and Veterinary Medicine, Changsha 410205, China; zhic2026@163.com

**Keywords:** ramie, corn silage, rumen, fermentation in vitro, methane

## Abstract

This study investigated the interactive effects of corn silage and ramie silage on in vitro rumen fermentation characteristics, aiming to provide a scientific basis and empirical evidence for the rational incorporation of ramie into ruminant diets. Four binary substrate mixtures were formulated based on dry matter (DM) mass ratios of corn silage to ramie silage: 100:0 (CON), 60:40 (R40), 20:80 (R80), and 0:100 (R100). Rumen fluid was collected from three adult Liuyang black goats surgically fitted with permanent rumen cannulas, and a standardized 48 h in vitro batch culture assay was conducted. Results demonstrated that increasing the proportion of ramie silage significantly decreased (*p* < 0.05) the DM degradation rate, neutral detergent fiber (NDF) degradation rate, acid detergent fiber (ADF) degradation rate, and total gas production per gram of substrate DM. Specifically, CON and R40 exhibited significantly higher values for all four parameters than R80 and R100 (*p* < 0.05). Methane production was significantly reduced in all ramie-containing treatments relative to CON (*p* < 0.05), whereas hydrogen production increased progressively with ramie inclusion level, with CON yielding significantly less H_2_ than both R80 and R100 (*p* < 0.05). Regarding fermentation parameters, increasing ramie proportion elevated (*p* < 0.05) both fermentation fluid pH and the acetate-to-propionate ratio, while total volatile fatty acid (TVFA) concentration declined linearly (*p* < 0.05). TVFA concentrations did not differ significantly between CON and R40, yet both were significantly greater than those in R80 and R100 (*p* < 0.05). Collectively, these findings indicate that ramie silage is a nutritionally valuable forage with potential as a high-quality partial replacement for conventional silages in ruminant feeding systems; however, its inclusion in corn–ramie mixed silages should not exceed 40% (on a DM basis) to maintain optimal fermentative efficiency and nutrient degradability.

## Simple Summary

Alfalfa—the world’s most widely cultivated protein-rich forage—performs poorly in the humid subtropical climate of southern China, contributing to chronic feed shortages in the region. In contrast, ramie (*Boehmeria nivea*), a native perennial crop with crude protein content exceeding that of conventional grasses, represents a locally adapted and nutritionally superior alternative. Existing feeding trials have demonstrated that ramie can fully substitute for alfalfa in dairy cow rations without adverse effects on milk yield or animal health. Nevertheless, corn silage remains the predominant energy source in ruminant diets across China, despite its inherently low protein concentration. Given their complementary nutritional profiles—ramie supplying high-quality protein and corn silage providing fermentable carbohydrates—their strategic combination holds potential to enhance dietary balance. Yet, no study to date has systematically investigated how blending ramie silage with corn silage modulates key rumen fermentation parameters or mitigates methane emissions. To address this gap, this study employs a standardized in vitro gas production system to quantify the effects of progressively replacing corn silage with ramie silage on rumen fermentation kinetics, volatile fatty acid profiles, and methane yield—thereby informing evidence-based, sustainable feed formulation strategies for livestock systems in southern China.

## 1. Introduction

High-quality forage constitutes the fundamental pillar for sustaining ruminant production performance and enabling the sustainable development of ruminant livestock systems. Alfalfa—a globally dominant high-quality leguminous forage—is poorly suited to large-scale cultivation in southern China due to its physiological intolerance to waterlogging and preference for warm, semi-arid climates. Consequently, persistent deficits in high-quality forage supply have severely constrained the growth and sustainability of ruminant husbandry in this region [1,2,3]. Addressing this constraint necessitates the identification and development of novel forage species adapted to the humid subtropical conditions of southern China. Ramie [2] emerges as a highly promising candidate: it exhibits exceptional agroclimatic adaptability across southern China, achieves high biomass yields, and contains substantially higher crude protein concentrations than conventional grasses—positioning it as a valuable, high-protein, non-conventional forage resource [3,4]. Notably, feeding trials with lactating Holstein cows demonstrated that partial or full substitution of alfalfa hay with ramie forage did not compromise milk composition, yield, or key hematological and metabolic parameters, thereby confirming its nutritional safety and practical feasibility in ruminant diets.

Within ruminant nutrition, corn silage serves as an irreplaceable source of fermentable fiber and energy. Its functional significance extends beyond energy provision: its physically effective fiber contributes critically to rumen motility and pH homeostasis, while its rapidly fermentable starch and structural carbohydrates support robust microbial growth and sustained volatile fatty acid (VFA) production—thereby underpinning both rumen health and host energy metabolism [5,6,7]. Nevertheless, corn silage is inherently deficient in crude protein (typically 6–8% DM), rendering it nutritionally inadequate as a sole roughage source for high-producing ruminants.

Despite the complementary nutritional profiles of ramie (high protein, moderate fiber digestibility) and corn silage (high energy, low protein), evidence-based strategies for their synergistic integration remain scarce. To date, no systematic in vitro or in vivo studies have evaluated how varying proportions of ramie silage and corn silage influence rumen fermentation dynamics—including VFA profile, ammonia-N concentration, microbial protein synthesis—and enteric methane mitigation potential. This study therefore employs a standardized in vitro gas production system to systematically investigate the effects of graded replacement of corn silage with ramie silage on rumen fermentation parameters and methane output. Findings will inform the rational formulation of dual-forage silage rations, advancing the sustainable intensification of ruminant production in southern China.

## 2. Materials and Methods

### 2.1. Experimental Materials and Design

Corn silage and ramie silage were produced from crops harvested in Yuanjiang City, Hunan Province, China. At harvest, plants were uniformly cut at a height of 70 cm to ensure consistent maturity and biomass composition. Following harvest, forage was chopped to a theoretical length of cut of 1–2 cm, packed into vacuum-sealed plastic bags (2 L capacity), and fermented under anaerobic conditions for 45 days at ambient temperature (22–28 °C) to complete ensiling. Representative subsamples (500 g fresh weight each) of corn silage and ramie silage were oven-dried at 65 °C for 48 h to constant weight, ground using a Wiley mill (Wiley Mill 4, Thomas Scientific, Swedesboro, NJ, USA) and passed through a 1 mm (40-mesh) stainless-steel sieve (UTS2040, Unitfine, Xinxiang, China). Ground samples were stored in airtight aluminum-laminated bags at −20 °C until chemical analysis and in vitro incubation.

Four dietary treatments were formulated based on dry matter (DM) proportions of corn silage and ramie silage, as presented in Table 1: 100:0 (CON, control), 60:40 (R40), 20:80 (R80), and 0:100 (R100). Each treatment included 2 biological replicates per batch, and 3 independent fermentation batches were conducted. Each in vitro incubation was conducted for 48 h under standardized anaerobic conditions.

### 2.2. In Vitro Fermentation

Three adult Liuyang black goats surgically fitted with permanent rumen cannulas were selected as rumen fluid donors. The goat basal diet composition (air-dry basis) included corn (22.00%), soybean meal (15.20%), wheat bran (10.00%), rice straw (50.00%), salt (0.80%), and premix (2.00%), with nutritional levels of digestible energy of 11.54 MJ/kg and a crude protein percentage of 14.30%. On the day of the experiment, fresh rumen fluid was collected via the cannula prior to morning feeding. For each goat, the collected fluid was immediately transferred into a separate pre-warmed (39.5 °C) thermos flask under a CO_2_ atmosphere and gently mixed to ensure homogeneity. The fluid from each goat was then filtered through six layers of sterile cheesecloth to remove large feed particles and kept anaerobically at 39.5 °C until use. Artificial rumen buffer solution was prepared according to the method of Menke et al. [8]. After addition of resazurin sodium indicator (0.2 mL of 0.2% *w*/*v* aqueous solution), the buffer was gassed with high-purity CO_2_ for 15 min to establish strict anaerobiosis—confirmed by complete decolorization of resazurin—and then equilibrated at 39.5 °C in a water bath. This constituted the artificial rumen incubation medium.

Exactly 0.6 g (dry matter basis) of each ground substrate was weighed into 150 mL serum bottles. To each bottle, 60 mL of the pre-equilibrated incubation medium was added, comprising 15 mL of strained rumen fluid and 45 mL of buffered mineral solution. All bottles were sealed with butyl rubber stoppers and aluminum crimps and incubated anaerobically in a shaking water bath (100 rpm) at 39.5 °C for 48 h. Three independent fermentation batches were conducted; within each batch, rumen fluid from the three goats was processed separately, and each treatment had two technical replicates per goat. Concurrently, three blank control bottles containing only 60 mL of the incubation medium (no substrate) were included per batch to correct for background gas production derived from endogenous microbial activity.

### 2.3. Sample Analysis

Gas pressure within each fermentation bottle was continuously monitored in real time via a stainless-steel tubing system connected sequentially to a three-way solenoid valve and a high-precision digital pressure transducer. Pressure data were acquired and logged by a computerized data acquisition system at 1 min intervals. Cumulative gas production was calculated using the ideal gas law, corrected for temperature, atmospheric pressure, and water vapor pressure, according to the method of Wang et al. [9]. The solenoid valve was automatically triggered by the data acquisition software when intra-bottle pressure exceeded 9 kPa, releasing headspace gas into a gas-tight sampling loop. The released gas was then automatically injected into a gas chromatograph (GC) equipped with a thermal conductivity detector (GC7890A, Agilent, Santa Clara, CA, USA) and a Porapak Q (19091P-QO3PT, Agilent, Santa Clara, CA, USA) column for simultaneous quantification of H_2_ and CH_4_ concentrations.

At the end of the 48 h incubation, the pH of the fermented fluid was measured immediately using a calibrated benchtop pH meter equipped with a glass electrode. Substrate dry matter (DM), organic matter (OM), crude protein (CP), neutral detergent fiber (NDF), acid detergent fiber (ADF), volatile fatty acids (VFAs), and ammonia nitrogen (NH_3_-N) were analyzed following standardized protocols described by Ran et al. [10]. The concentrations of VFAs and NH3-N were determined using gas chromatography (GC7890A, Agilent, Santa Clara, CA, USA) and a spectrophotometer (UV-2600, Shimadzu Global Laboratory Consumables Co., Ltd., Shanghai, China), respectively.

### 2.4. Data Calculation and Statistical Analysis

The potential maximum gas production, gas production rate, and initial substrate degradation rate were estimated following Wang et al. [11]:

GPt = Vf [1 − EXP(−kt)]/[1 + exp [1 + exp(b − kt)]

FRD0 = k/[1 + exp(b)]

where GPt represents gas production at time point t; Vf is the final asymptotic gas volume (mL); k represents the fractional rate of gas production; b is a constant of the gas production curve; t represents incubation time (h); and FRD0 represents the initial fractional rate of degradation at t = 0.

Statistical analyses were performed using SPSS software (version 21.0; IBM Corp., Armonk, NY, USA). A linear mixed model was fitted to the data, with treatment as the fixed effect, and fermentation batch and replicates within each batch as random effects. Differences were considered statistically significant at *p* < 0.05.

## 3. Results

### 3.1. Effects of Corn Silage–Ramie Silage Combinations on In Vitro Ruminal Fiber Degradation

As presented in Table 2, increasing the inclusion level of ramie silage in the substrate significantly reduced the in vitro degradation of DM, NDF, and ADF (*p* < 0.01). The CON group showed significantly greater DM, NDF, and ADF degradation than both the R80 and R100 groups (*p* < 0.05), but did not differ significantly from the R40 group (*p* > 0.05).

### 3.2. Effects of Corn Silage–Ramie Silage Combinations on In Vitro Ruminal Gas Production Kinetics

As presented in Table 3, the inclusion level of ramie silage did not significantly affect the gas production rate or initial substrate degradation rate (*p* > 0.05). In contrast, gas production per gram of substrate, gas production per gram of degraded substrate, and potential maximum gas production all declined with increasing ramie silage proportion. The CON group showed significantly greater values for gas production per gram of degraded substrate and potential maximum gas production than those of the other three groups (*p* < 0.05), and gas production per gram of substrate was higher than that of the R80 and R100 groups (*p* < 0.05).

### 3.3. Effects of Corn Silage–Ramie Silage Combinations on In Vitro Ruminal Methane and Hydrogen Production

As presented in Table 4, the inclusion level of ramie silage did not significantly affect methane or hydrogen production rates (*p* > 0.05). However, methane concentration, gas production per gram of substrate, and potential maximum gas production all declined with increasing ramie silage proportion, and CON exhibited significantly greater values than those of the R40, R80, and R100 groups (*p* < 0.05). In contrast, hydrogen concentration, gas production per gram of substrate and potential maximum gas production all increased with increasing ramie silage proportion, and CON showed significantly lower values than those of the other three groups (*p* < 0.05).

### 3.4. Effects of Corn Silage–Ramie Silage Combinations on In Vitro Ruminal Fermentation and Volatile Fatty Acid Profile

As presented in Table 5, inclusion of ramie silage did not significantly affect ammonium nitrogen concentration in the fermentation fluid (*p* > 0.05). In contrast, both pH and the acetate-propionate ratio increased linearly with increasing ramie silage proportion (*p* < 0.01), and CON exhibited a significantly lower pH than the R40 and R100 groups (*p* < 0.05). Total volatile fatty acid (TVFA) concentration declined, with CON showing significantly greater TVFAs than both the R40 and R100 groups (*p* < 0.05). Moreover, increasing ramie silage proportion decreased the molar proportions of propionate and butyrate (*p* < 0.01) while increasing those of acetate, isobutyrate, valerate, and isovalerate (*p* < 0.01).

## 4. Discussion

### 4.1. Effects of Corn Silage–Ramie Silage Combinations on In Vitro Ruminal Fiber Degradation

Rumen fermentation efficiency is a core indicator of feed digestibility and utilization, directly reflecting the capacity of ruminal microbiota to degrade dietary substrates [12,13]. Fibrolytic bacteria—particularly those synthesizing cellulases, xylanases, and other cell-wall-degrading enzymes—play a pivotal role in ruminal fiber degradation and thereby support efficient utilization of fibrous feed resources by ruminants [10]. These enzymes catalyze the breakdown of complex fiber structures into fermentable monomers, which are further metabolized to produce energy substrates for ruminants. Among dietary components, fiber is the primary determinant of ruminal digestion kinetics; consequently, fiber degradation rates serve as quantitative proxies for the extent of ruminal fiber digestion, providing a reliable metric to evaluate rumen fermentation performance and feed nutritional value.

In this study, ramie silage contained higher concentrations of CP and ADF than corn silage; thus, increasing the ramie silage inclusion level increased both CP and ADF concentrations in the mixed diets. Consistent with this compositional shift, neutral detergent fiber digestibility (NDFD) and acid detergent fiber digestibility (ADFD) both declined significantly with increasing ramie silage proportion. These results are consistent with those of Tian et al. [14], who observed decreased in vitro dry matter degradability (DMD) and NDF degradability upon replacing 60% of corn silage with ramie silage in goat diets. This decline is likely attributable to ramie’s inherently high lignin content, which forms recalcitrant lignin–cellulose–hemicellulose complexes that restrict enzymatic hydrolysis; consequently, Calabro et al. [15] reported that substrates with higher lignin content result in reduced DM degradability; moreover, ramie contains appreciable levels of condensed tannins, which directly inhibit fibrolytic enzyme activity and impair substrate degradation.

### 4.2. Effects of Corn Silage–Ramie Silage Combinations on In Vitro Ruminal Gas Production

In vitro ruminal gas production and its kinetic parameters are core indicators for characterizing feed fermentation profiles and quantifying the extent of ruminal utilization of dietary nutrients. Greater total gas production is generally indicative of higher substrate fermentability in the rumen [16]. In this study, gas production per gram of substrate decreased significantly when ramie silage inclusion exceeded 40%. Similarly, Tian et al. [17] conducted in vitro fermentation experiments using binary mixtures of forage ramie and alfalfa at varying dry matter ratios to evaluate their effects on two key fermentation parameters: gas production per gram of substrate and potential maximum gas production. Results indicated that both gas production per gram of substrate and potential maximum gas production declined with increasing ramie inclusion level. Notably, when ramie constituted more than 40% of the dry matter, gas production per gram of substrate decreased significantly (*p* < 0.05) relative to mixtures with lower ramie proportions. These results demonstrate that robust evaluation of unconventional feedstuff necessitates integrated analysis of both endpoint gas yield and kinetic parameters—including potential maximum gas production, fractional degradation rate, and lag time—to fully characterize their ruminal fermentation behavior.

CH_4_ production is strictly dependent on the abundance and metabolic activity of methanogenic archaea (methanogens); high dietary fiber concentrations have been shown to suppress methanogen activity and proliferation [18,19]. Ramie contains appreciable levels of condensed tannins, which delay microbial degradation of fibrous substrates and concurrently inhibit CH_4_ synthesis [20]—a finding consistent with prior studies that link condensed tannin-rich forages to reduced ruminal methanogenesis. Under the present experimental conditions, increasing ramie silage proportion significantly reduced CH_4_ emission, which may be closely associated with the combined effects of its high fiber content and condensed tannins on rumen methanogen communities.

Research reports have also shown that the total phenolic compound content in ramie ranges from 0.52 to 2.41 mg/g DW, while the total flavonoid content varies between 0.40 and 2.50 mg/g DW [21]. Previous studies have demonstrated that when forage ramie is mixed with sweet sorghum for in vitro fermentation, the methane production of the system decreases with an increasing proportion of added forage ramie [22]. This inhibitory effect can be attributed to the rich flavonoids in ramie: these active substances can directly or indirectly regulate the activity of rumen microorganisms, or act on enzymes associated with methane synthesis (e.g., methane synthase), thereby suppressing the methane generation process [23]. Additionally, flavonoids can inhibit methane production-related signal transduction by interfering with the synthesis, release, or receptor binding of signal molecules, and regulate the transcriptional and expression levels of related genes by binding to transcription factors or acting on gene promoter regions, thereby altering the activity of key enzymes or the concentration of key substrates in the methane generation pathway [24]. It should be noted that the inhibitory effect of flavonoids on methane production is heterogeneous, which may vary depending on flavonoid types, action concentrations, and environmental conditions, as well as the types and physiological states of the test organisms. Therefore, further in-depth analysis is still required to clarify the specific mechanism by which the bioactive components of ramie inhibit methane production.

H_2_ serves as the obligate precursor for CH_4_ synthesis by methanogens and a central intermediate in VFA formation; most ruminal H_2_ is normally consumed by methanogens to produce CH_4_ [25]. Ruminal H_2_ partial pressure is maintained under dynamic equilibrium, and either reduced H_2_ generation or impaired interspecies H_2_ transfer suppresses methanogen activity, thereby lowering CH_4_ output [26]. In this study, elevated H_2_ accumulation coincided with decreased CH_4_ production, indicating compromised H_2_ utilization by methanogens. This implies that ramie silage substitution disrupted syntrophic H_2_ transfer between fibrolytic bacteria and methanogens—most likely via tannin-induced inhibition of methanogen enzymatic function or interference with physical cell–cell interactions—resulting in net H_2_ accumulation.

### 4.3. Effects of Corn Silage–Ramie Silage Combinations on In Vitro Ruminal Fermentation Parameters

Rumen pH is a direct indicator of ruminal environmental stability and a key regulator of microbial growth, proliferation, and metabolic activity; it also significantly influences both the concentration and molar distribution of VFAs [27]. In this study, ruminal pH increased significantly across all ramie silage-supplemented groups yet remained within the physiologically normal range of 6.5–6.8. This rise aligns with the concurrent decline in TVFA concentration, as reduced production of acetate and propionate—major contributors to ruminal acid load—lowers net hydrogen ion generation and thereby promotes mild alkalinization. Similar results have also been observed in animal experiments. In one study, the dietary ramie dosage for dairy cows was gradually increased, and rumen fluid was analyzed. It was found that the rumen fluid pH value increased with the elevation of dietary ramie dosage [2].

NH_3_-N is a core metabolite in rumen nitrogen metabolism and the main nitrogen source for microbial protein (MCP) synthesis. Maintaining an appropriate ammonia nitrogen concentration is crucial for the efficient synthesis of microbial protein and the promotion of microbial growth [28]. Rumen microorganisms hydrolyze dietary protein into free amino acids, small peptides, and ammonia, which combine with carbon skeletons derived from VFAs and carbon dioxide to form microbial protein [29]. In this experiment, there was no significant difference in ammonia nitrogen concentration among all treatment groups, indicating that the gradual replacement of corn silage with ramie silage can maintain sufficient nitrogen supply to support microbial protein synthesis without affecting rumen nitrogen metabolism balance.

The concentration and molar proportions of VFAs are core metrics for characterizing ruminal fermentation patterns and constitute the principal energy source for ruminants. Acetate is predominantly generated by fibrolytic bacteria during degradation of cellulose and hemicellulose in plant cell walls, whereas propionate arises primarily from starch- and sugar-fermenting bacteria metabolizing soluble carbohydrates [30]. In this study, the molar proportion of acetate in total VFAs increased with increasing ramie silage inclusion, consistent with in vivo findings reported by Gao et al. [2]. Because acetate is directly absorbed and oxidized by peripheral tissues—thereby sparing endogenous fat mobilization—a higher acetate proportion reflects a fermentation profile conducive to efficient host energy capture. Multiple studies demonstrate that ramie, particularly when co-fermented with other forages, consistently elevates acetate molar proportion while suppressing CH_4_ emission [17]; the present results corroborate this pattern. However, the precise mechanisms—including potential shifts in fibrolytic community composition, altered interspecies H_2_ transfer kinetics, or tannin-mediated modulation of methanogen enzyme activity—are yet to be elucidated through targeted mechanistic studies.

## 5. Conclusions

Under the present in vitro experimental conditions, graded replacement of corn silage with ramie silage increased dietary CP concentration and decreased CH_4_ emissions; however, substitution beyond 40% compromised key ruminal fermentation parameters—including NDFD, TVFA concentration, and acetate molar proportion—indicating that 40% represents the upper threshold for maintaining efficient ruminal fermentation.

## Figures and Tables

**Table 1:** Nutrient levels of the fermentation substrate (DM basis).

Items	CON	R40	R80	R100
Ingredient				
Corn silage	100	60	20	0
Ramie silage	0	40	80	100
Chemical composition				
Dry matter, %	37.44	36.80	36.15	35.83
Organic matter, %	93.42	85.71	78.00	74.14
Crude protein, %	8.16	11.68	15.19	16.95
Neutral detergent fiber, %	67.04	62.66	58.28	56.09
Acid detergent fiber, %	38.93	39.51	40.09	40.38
Gross energy/MJ·kg^−1^	14.50	13.99	13.48	13.23

**Table 2:** Effects of corn silage–ramie silage combinations on in vitro ruminal fiber degradation (DM basis) %.

Items	CON	R40	R80	R100	SEM	*p* Value
Dry matter digestibility	40.36 a	36.43 a	27.69 b	25.01 b	2.29	0.001
Neutral detergent fiber digestibility	42.89 a	39.86 a	31.06 b	22.69 c	0.76	<0.001
Acid detergent fiber digestibility	35.78 a	34.45 a	25.59 b	19.53 c	0.94	<0.001

a, b, c Means within rows with different super script letters differ (*p* < 0.05).

**Table 3:** Effects of corn silage–ramie silage combinations on in vitro ruminal gas production kinetics (DM basis).

Items	CON	R40	R80	R100	SEM	*p* Value
Gas production per gram of substrate (mL/g)	202.15 a	174.56 a	139.37 b	100.76 c	4.40	<0.001
Gas production per gram of substrate degradation (mL/g)	520.52 a	439.37 b	419.31 b	352.76 c	20.10	0.015
Potential maximum gas production (mL/g)	232.08 a	184.04 b	138.62 c	136.34 c	12.33	0.002
Production rate (h^−1^)	0.04	0.03	0.03	0.02	0.59	0.261
Initial substrate degradation (h^−1^)	0.02	0.02	0.02	0.02	0.28	0.791

a, b, c Means within rows with different super script letters differ (*p* < 0.05).

**Table 4:** Effects of corn silage–ramie silage combinations on in vitro ruminal methane and hydrogen production (DM basis).

Items	CON	R40	R80	R100	SEM	*p* Value
Methane production						
Methane contents (%)	16.49 a	11.10 b	8.66 c	4.17 d	0.14	<0.001
Gas production per gram of substrate (mL/g)	31.56 a	23.21 b	14.96 c	10.29 d	0.47	<0.001
Potential maximum gas production (mL/g)	36.18 a	26.28 b	17.93 c	16.60 c	19.56 c	<0.001
Production rate (h^−1^)	0.08	0.10	0.10	0.09	0.01	0.563
Hydrogen production						
Hydrogen contents (%)	0.01 c	0.03 c	0.08 b	0.13 a	0.01	<0.001
Gas production per gram of substrate (mL/g)	0.03 d	0.07 c	0.14 b	0.19 a	0.01	<0.001
Potential maximum gas production (mL/g)	0.03 d	0.08 c	0.16 b	0.19 a	0.02	<0.001
Production rate (h^−1^)	0.31	0.32	0.41	0.28	0.08	0.929

a, b, c, d Means within rows with different super script letters differ (*p* < 0.05).

**Table 5:** Effects of corn silage–ramie silage combinations on in vitro ruminal fermentation and volatile fatty acid profile (DM basis).

Items	CON	R40	R80	R100	SEM	*p* Value
pH	6.51 c	6.64 b	6.72 ab	6.78 a	0.02	<0.001
Ammonia (mmol/L)	17.87	18.88	18.35	16.88	0.70	0.307
Acetate–propionate	2.24 d	2.46 c	2.73 b	2.86 a	0.02	<0.001
TVFA (mmol/L)	94.21 a	91.12 a	83.26 b	76.65 c	1.12	0.001
The percentage of individual VFAs (%)						
Acetate	61.13 d	63.26 c	65.00 b	65.46 a	0.11	<0.001
Propionate	27.43 a	25.79 b	23.84 c	22.90 d	0.16	<0.001
Butyrate	8.69 a	7.83 b	7.64 b	7.77 b	0.08	<0.001
Isobutyrate	0.78 d	0.94 c	1.06 b	1.17 a	0.02	<0.001
Valerate	0.82 d	0.86 c	0.95 b	1.03 a	0.01	<0.001
Isovalerate	1.15 d	1.32 c	1.51 b	1.67 a	0.03	<0.001

a, b, c, d Means within rows with different super script letters differ (*p* < 0.05).

## Data Availability

The data will be made available from the corresponding author upon reasonable request.
